# Support staff liaising effectively with professionals for the rational use of psychotropics for behaviours that challenge in adults with intellectual disabilities: Findings from a co-design event

**DOI:** 10.3389/fpsyt.2022.954522

**Published:** 2022-09-29

**Authors:** Bharati Limbu, Shoumitro Shoumi Deb

**Affiliations:** Department of Brain Sciences, Faculty of Medicine, Imperial College London, London, United Kingdom

**Keywords:** Experience Based Co-Design, co-production, people with intellectual disabilities, behaviours that challenge, psychotropic medications, psycho-education programme, interdisciplinary collaboration

## Abstract

Experience Based Co-Design (EBCD) and co-production are interdisciplinary collaborative approaches to improve health care services by involving all stakeholders. These approaches capture the experiences of all stakeholders who come in contact with services and use experiences as evidence to promote and implement service changes. The use of psychotropic medications for behaviours that challenge (BtC) in people with intellectual disabilities (ID) is a complex issue because of its off-licence use and use in combination with other medications for physical and psychiatric co-morbidities, which leads to overmedication of people with ID. As support staff plays a pivotal role in the prescribing for people with ID, we have developed a staff training programme, SPECTROM, to help reduce overmedication. A project team developed SPECTROM under the guidance of a Programme Development Group (PDG) consisting of 21 stakeholders. The PDG analysed data from a literature review, four focus groups and a co-design event day involving 26 stakeholders. In this paper, we have presented data based on the findings from the co-design event day, primarily on the issue of support staff effectively liaising with professionals such as doctors, nurses, and other community learning disability team members. In-depth information and recommendations were proposed at the co-design event, which helped develop the draft SPECTROM. The draft was finalised after receiving feedback from 56 stakeholders. Co-production and a modified EBCD can be successfully used to create training interventions and improve health care services. More research should utilise co-production and EBCD and use service users’ experiences to develop interventions and improve health care services.

## Introduction

Experience Based Co-Design (EBCD) is an interdisciplinary collaborative approach to improving healthcare services by enabling service users, caregivers, and professionals to collaborate and co-design better services. EBCD uses stakeholders’ experiences as evidence to improve service users’ experience and health care services. The stakeholders play an active role by directly contributing to the design or change of services. Its crucial feature is equal close collaboration among all stakeholders. It was first piloted in a Head and Neck Cancer service (1). Subsequently, a toolkit was developed by the King’s Fund (2). Since then, this method has been used in different countries and different healthcare settings to improve patient experience and design better healthcare services. EBCD has the following stages: setting up the project, gathering experiences of patients and staff, co-design events, and reviewing and generating a consensus (1). The co-design method uses participatory experience tools to collect and reflect people’s experiences and facilitate quality improvements during the co-design event. Participatory design exercises or tools will help identify “touch points” or critical moments, which are defining moments associated with emotional connections when people come into contact with services (3). This will help develop and improve services based on experiences (4).

**FIGURE 1:**
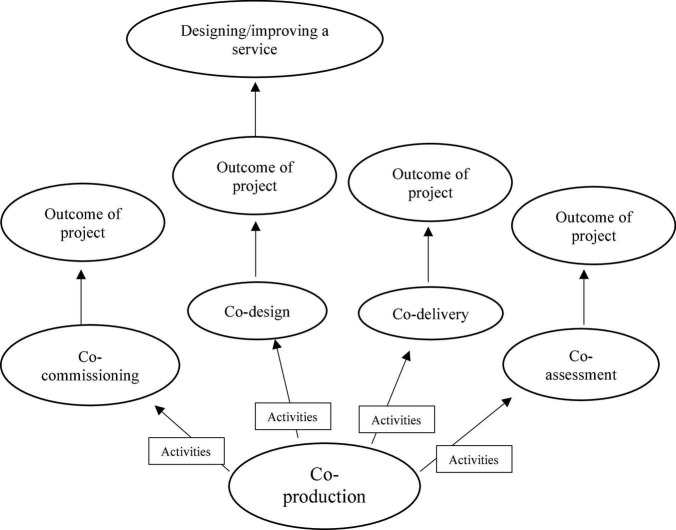
Types of co-concepts in co-production projects.

Co-design, an essential part of EBCD, is also a type of co-production (see Figure 1). The activities based on co-production projects’ outcomes will dictate the type of “co” concepts. Co-production can lead to co-commissioning, co-design, co-delivery and co-assessment of public service. Co-design is the process of designing a service in collaboration with stakeholders (5). Where co-production can involve public and service users for various outcomes and reasons, even outside of health care, EBCD is focused on improving health care service, emphasising designing experiences collaboratively. EBCD has been successfully applied in services for people with intellectual disabilities (ID) and vulnerable populations, caregivers and professionals to co-produce and co-design services for various outcomes (6–8). In Unwin et al. (7) study, EBCD was used to collaborate with people with ID who were of ethnic minorities (Black and Asian) to develop tools to facilitate culturally-sensitive communication and information sharing, service planning and delivery.

Psychotropic medications are healthcare interventions often prescribed for Behaviours that Challenge (BtC) in people with ID. Examples of BtC are aggression and self-injury, which may be manifested by around 22% of people with ID (9). However, they are often prescribed outside of their licence (10) and with medications for other health or psychiatric conditions (9). Thus, this leads to the overmedication of people with ID. According to Public Health England, approximately 35,000 adults with ID take psychotropic medications daily off-licence (11). This is concerning as psychotropic medications are also often prescribed at a higher than the recommended dose, and polypharmacy is common (12).

There are vast differences in perceptions of psychotropic medication use for BtC across disciplines. Prescribers believe they have an essential role in ensuring the appropriate prescription of psychotropic medications for BtC but stress the importance of multidisciplinary collaboration, which is often lacking (13, 14). When family caregivers were consulted, they believed psychiatrists or prescribers were “the expert” and often felt left out from important decisions. Family caregivers believe the care of people with ID can be improved. They felt holistic management of BtC was lacking and felt marginalised with no information or influence (15, 16). People with ID were compliant with psychotropic medications prescribed for BtC despite experiencing side effects and having limited knowledge of the impact or benefits of psychotropic medications (15, 17). They were unaware of their rights to be involved in medication decisions and often placed unwavering faith in the doctors’ authority and expertise (15). Majority of people with ID were not informed about their psychotropic medication and the reason for the prescription (17). Therefore, effective communication and liaison with professionals and holistic and multidisciplinary collaboration are lacking. There is a desire for patients, professionals, support staff, and family caregivers to reduce overmedication and a need for a platform to collaborate so that all these views can be taken forward to reduce the overmedication of psychotropic medication for BtC.

The authors recently developed a training programme called SPECTROM (Short-term Psycho-Education for Carers To Reduce Over Medication of people with intellectual disabilities) for support staff to help reduce the overmedication of people with ID (18). Support staff plays a pivotal role in the prescribing process (19, 20). Support staff is often present during initiation of psychotropic medication or support people with ID to medication reviews. Staff training and organisational policies are also crucial factors in the success of withdrawal of psychotropic medications (21). Staff training is therefore essential in reducing overmedication in people with ID. This paper describes the methodology used in developing SPECTROM and the findings of the co-design event, particularly one theme called effective liaison with professionals. Further details of the method to develop SPECTROM were presented in a separate paper (22).

## Materials and methods

### Phases of Experience Based Co-Design

**FIGURE 2:**
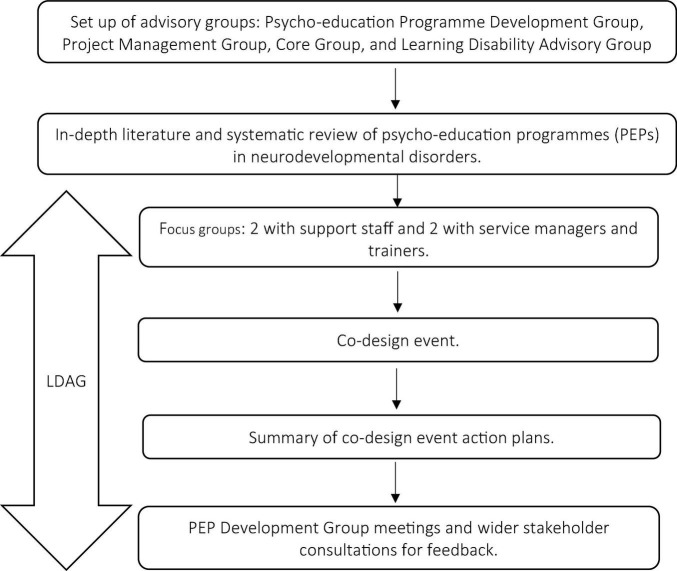
Modified Experience Based Co-Design (EBCD) methodology for the development of SPECTROM training.

SPECTROM was developed according to Medical Research Council’s guideline for developing and evaluating the complex intervention (23) and a modified EBCD (see Figure 2) (1). The modified version of EBCD had four stages as part of the EBCD process. In phase one, a Programme Development Group (PDG), consisting of 21 stakeholders, a Project Management Group comprising 18 stakeholders, a Core Project Group, and a Learning Disability Advisory Group (LDAG) were set up. In phase 2, in-depth literature reviews and focus groups were held to gather literature-based evidence and understand the stakeholders’ experiences. Opinions on medication use for BtC and recommendations for the format and content of SPECTROM were collated. Then a co-design event was held informed by the focus groups’ findings to gather a consensus on the structure, content and delivery of SPECTROM, which was further ratified by the PDG and finalised after receiving feedback from 56 stakeholders.

### Participants

All stakeholders involved were invited to attend a co-design event held in July 2019 at a conference centre in London, UK. Stakeholders included service managers, support staff, trainers, Community Learning Disabilities Team (CLDT) members, consultant psychiatrists, family caregivers and people with ID. Initially, invites were sent to everyone involved in the SPECTROM project and colleagues that expressed interest in the SPECTROM project because it was discussed that it might be challenging to gather enough attendees for the event. Everyone invited to the co-design event was asked to confirm attendance *via* email. All correspondence in this study was made by email.

Family caregiver organisation, the Challenging Behaviour Foundation (CBF), was asked to send invites on behalf of the SPECTROM project team to family caregivers. Service provider organisations involved in the project (Achieve Together, AT-Autism, Avenues Group, Dimensions-UK, Mencap, Milestones Trust, and National Autistic Society) identified service/house managers who were available and happy to participate in the project. The service managers were then asked to identify support staff for the co-design event. Support staff was selected based on availability. Out of 80 invites, 31 stakeholders confirmed attendance. This included five service managers, five support staff, six trainers, five CLDT members, four psychiatrists, and six family carers. As we wanted the perspective of all stakeholders, one participant from each stakeholder category was included to form a group. In total, five groups were constructed for the co-design event, each compromising one service manager, support staff, trainer, CLDT member, psychiatrist and family carer where possible. The primary purpose of the co-design was to develop the SPECTROM training programme. Therefore, we ensured a Positive Behaviour Support (PBS) trainer was included to exploit their expertise and ensure no overlap with any existing training for support staff. The authors formed part of the core team members who floated around all groups to provide support, answer queries, and encourage engagement, interaction and discussion on the co-design event. Other stakeholders, such as general practitioners and pharmacists, were part of the project group and PDG but did not attend the co-design event.

A community nurse who was the facilitator of Cornwall Learning Disability Advisory Group (LDAG) and part of the project team took part in the co-design event day to represent the views of people with ID. The LDAG included 12–14 adults with ID who could communicate, some of whom displayed BtC in the past for which they received psychotropic medication. Some have gone through the withdrawal of these medications recently. The main points from the co-design event day were presented in an accessible format by the facilitator to LDAG members in their next meeting. The meeting format was informal, and no structure like the co-design event was employed. The feedback from LDAG was brought back to the project team, who incorporated these ideas into the module themes.

### The development of activities

As part of the SPECTROM study, two focus groups were conducted: one with support staff only (*n* = 8) and one with service managers and trainers (*n* = 8). The first focus group gathered their opinions on the use of psychotropic medication for BtC, views related to BtC, and the relationship between BtC and psychiatric disorders. The second focus group gathered participants’ opinions on SPECTROM training content and format. The focus group discussions were recorded and analysed using thematic analysis with the help of NVivo software (24). The themes were then identified and used as topics in the co-design event. The themes and analysis process were overseen and verified by two experienced qualitative researchers involved in the SPECTROM project. More information on the thematic analysis of focus groups can be found in a separate paper (25). The topics identified included autism spectrum disorder (ASD) and attention deficit hyperactivity disorder (ADHD), staff attitude, effective liaison with family caregivers, effective liaison with professionals (health professionals-General Practitioners (GPs), CLDT members, psychiatrists, pharmacists), information for a medication review, communication and interaction issues between caregivers and people with ID, care staff empowerment, communication issues-behaviour as a means of communication, engaging people with ID effectively, and care staff reaction to BtC, fear/stress-self-reflection, assessment, and management. Participants were asked to prepare content for each theme by completing tasks as activities for the co-design event.

A guide (crib sheet) was developed for each theme to instruct participants on how to complete the activities. The guide informed participants of the allocated theme/topic for discussion, how much time each group had to discuss the issue, provided information on the possible format of the training programme and its target audience, and provided five steps/tasks for the event day. The first task provided participants with example content for each theme and asked participants of each group to add content titles, case vignettes and texts associated with each content. The guide also included quotes from the focus groups to provide participants with suggestions for content, case vignettes and text. Participants were asked to complete other tasks in digital order if time allowed. Task 2 asked participants to recommend how the contents of the allocated topic module can be implemented in day-to-day practice. Task 3 asked participants to rank the contents they produced from basic to specific and detailed to cater to trainees with different experience and expertise levels. Task 4 asked participants to recommend ways the written modules can be linked to the face-to-face training session and task 5 asked participants to contribute to the contents/case vignettes for other themes if they had spare time. An example of the guide can be found in Supplementary material 1.

### Co-design event

On the day of the co-design event, attendees were registered and given their allocated group numbers. We provided participants hand-outs on the event’s agenda and identified themes from both sets of focus groups, themes for discussion, and guidelines for the day’s activities. Additionally, views of people with ID on BtC and its management based on a Dutch study by Wolkorte et al. (26) provided some suggestions on texts for the contents (see Supplementary material 2). The co-design event started with a PowerPoint presentation for 45 min to provide background information, the aims of the SPECTROM project, the day’s structure, and set purposes for the day. Attendees were also shown a video of a successful antipsychotic withdrawal and its positive effects on the person with ID. Attendees were given 1 h to work on their activities and 5 min to present their group work. Each group was assigned a flip chart to write their suggestions and recommendations and asked to choose a spokesperson to present their work. One author (BL) recorded the field notes, and the second author (SD) verified them. The event lasted 6 h, including time for breakfast and lunch. There were two sessions, morning and afternoon, to cover ten potential topics. Each group received one theme for the morning session and one for the afternoon session. The flip chart papers and any paperwork with suggestions made by the participants were gathered at the end of the event. These and the recorded suggestions were used to develop an action plan for SPECTROM content and format. Attendees were also encouraged to email any comments or further suggestions if there was no room for discussion.

Of the 31 who agreed to attend, 26 participants participated in the co-design event. One family carer, two support staff, one trainer, and one professional (CLDT member) could not participate in the co-design event. The flip chart papers and notes taken during the presentation of the co-design event were used to formulate SPECTROM training modules and materials (see Supplementary material 3 for an example flip chart). Rich, detailed information was gathered during the co-design event. All suggestions and recommendations of stakeholders were recorded. A summary of key points for each theme was developed to generate action plans.

## Results

SPECTROM resources include internal and external resources that are hyperlinked with other websites that provide relevant information. Internal resources include (a) accessible information on 32 commonly used psychotropic medications for people with ID, (b) Comprehensive Assessment of Triggers for behaviours of concern Scale (CATS), and (c) a patient/family caregiver-held Yellow passport that provides all relevant information, including health information in an accessible format which could be shared among various services as a need-to-know basis. There are two core modules, (a) Medication and (b) Alternatives to medication (ATM). The two core modules are used for face-to-face workshops through which other modules are introduced. There are 12 additional modules, (1) Medication review, (2) Medication withdrawal review, (3) Assessment of behaviour, (4) Effective liaison with family carers and advocates, (5) Effective liaison with professionals (GP, CLDT members and psychiatrists), (6) ATM– introduction, (7) Communication needs, (8) Effective engagement with people who have intellectual disabilities, (9) Physical disorders and challenging behaviour, (10) Psychiatric disorders and challenging behaviour, (11) Autism (ASD), and (12) Attention deficit hyperactivity disorder (ADHD) (https://spectrom.wixsite.com/project).

Due to lack of space and the amount of data gathered, we have presented in this paper data only on the theme, “effective liaison with professionals (health professionals-GPs, CLDT members, psychiatrists, pharmacists).” Data from other themes will be presented in separate papers.

This theme/topic was related to understanding support staff’s (and also the person with ID and their family caregivers) experience of liaising with any health professionals and how this could be improved based on their previous knowledge of communicating with health professionals. Health professionals (psychiatrists and CLDT members) in the groups were asked to draw from their experience of communicating with support staff, people with ID and their family caregivers and recommend how the liaison between professionals and support staff, and people with ID and their families could be improved.

### Knowledge is power gather up-to-date information about the person with intellectual disabilities

Participants stated, “knowledge is power.” They suggested that support staff should prepare thoroughly for professional appointments by learning and knowing about the person with ID they are accompanying to appointments. This includes understanding the background of people they support, including medication history and key events or trauma people with ID have experienced or may experience. Support staff should understand “any clinical or physical health conditions/diagnoses” the individuals have. This will help support staff understand reasons for BtC and differentiate BtC that were triggered by key events from those that occurred due to medication or changes in medication etc. This allows support staff to communicate correctly to professionals the reasons for changes in BtC and medications’ impact on BtC.

Participants recommended that true collaboration should put the person with ID and their families at the centre, and the SPECTROM training should promote this theme. Support staff should increase their knowledge about the person with ID, such as gathering information about their communication needs, likes and dislikes, support needs, support plans, health action plans, behaviour support plans, etc. This will allow support staff to be adequately informed and convey the correct information to the professional during meetings and appointments. Having the knowledge will empower support staff and help to address any queries from the professionals or challenge any decision that is not in the person’s best interests.

Participants also suggested that SPECTROM training should create a checklist for support staff to gather documents and information needed for each appointment. These may include monitoring and preparing data charts such as seizure charts, Antecedent-Behaviour-Consequence (A-B-C) charts, Medication Administration Record (MAR sheet), etc. It is also essential to keep the person’s health and communications passports up to date if it needs to be shared. Participants recommended that the consent to share sensitive information and the capacity to consent should be recorded properly. They also suggested that support staff or service managers contact professionals before appointments and agree on the required information. It was recommended that any critical documents/information should be shared with the professionals before the appointments, if possible. By providing the information and documents before the formal appointment, professionals will also have more time to interact with people with ID instead of spending time looking at the documents during the appointments.

### Identify a key support staff worker

Participants suggested that one way to ensure support staff knew the person with ID well was to allocate a key support staff worker who would attend all meetings and appointments as much as possible. The key support staff will gather up-to-date information about the person and share any required information, learn to ask the right questions and disseminate professionals’ recommendations on time to other stakeholders on a need-to-know basis. The key support staff should liaise with key professionals (psychiatrists/CLDT etc.), key workers from the CLDT, and a key family member. This will ensure the communication of the right information at the right time among the right people who care for the person with ID as well as the person with ID and their families.

### Informal in-house meetings

Another key recommendation was to encourage in-house informal meetings with the person with ID, their families and other professionals involved, and service managers/key staff (with the person’s consent). Participants suggested that the key support staff should discuss the checklist (of information and documents needed for the formal appointment) and the goal of the appointment in the informal meeting. This will also keep all stakeholders informed about issues discussed in the appointment.

### Preparing the person with intellectual disabilities for appointments

Another key recommendation was to prepare people with ID for their appointments using their preferred communication method (scripts, social story, desensitisation, and communication strategies).

### Questions to ask the professionals

Participants recommended that the SPECTROM module should provide examples of the type of questions support staff should ask prescribers or other professionals during an appointment.

### Other recommendations

Another key recommendation was that professionals such as GPs and psychiatrists should receive training on (a) how to support and communicate with people with ID and/or ASD during the appointment, (b) help support staff understand professional language and help them to be specific with questions, and (c) encourage support staff to request in advance reasonable adjustments to be made by professionals in the environment/clinic. These may include allowing more time, several short interviews than one long interview, using accessible information, allowing the person with ID to come with a familiar person or communication partner etc.

### Case study examples

Participants provided case vignettes for inclusion in the SPECTROM module. Examples are (a) annual health checks at home, (b) a case study around adjusting and simplifying communication, and (c) a case study in a hospital environment where PRN (Pro Re Nata, as needed) medications were prescribed without consulting with the person with ID/family, and (d) providing information and statistics on success and failures of psychotropic medication withdrawal to allay the fear of medicine reduction/withdrawal.

We developed the SPECTROM module, “an effective liaison with professionals,” by incorporating the recommendations from the co-design event. Summary recommendations in the module include (a) effective collaboration among the stakeholders is the key to successful care delivery, (b) identifying a key support staff, a key professional and a key family caregiver, and (c) key support staff should gather up-to-date information about the person and share with others on time, (d) information should be exchanged among the stakeholders before any multi-disciplinary meeting, (e) mutual understanding and respect to each other’s opinion are necessary to resolve differences in opinion, (f) always involve the person with ID and their family caregivers or an advocate in all discussions about the person with ID, (g) regular contact and information exchange are essential for providing good quality care, and (h) common purpose of the collaboration is to improve the quality of life of the person with ID.

## Discussion

This paper describes the development process of a support staff training resource, SPECTROM, and describes the findings of one of its themes/modules, “effective liaison with professionals.” The recommendations made by the participants indicate that effective liaison among stakeholders has not always been optimal and can be improved. Both professionals and support staff must take measures to improve effective liaison and achieve true collaboration to promote holistic care of people with ID. The recommendations are thus made for both support staff and professionals.

Key recommendations include (a) involving people with ID for discussions and decision making, (b) informing and preparing people with ID for appointments, (c) identifying a key support staff for each person with ID, and (d) understanding people with ID and gathering up to date information about them, (e) timely information sharing among all stakeholders, and (f) informal in-house staff-led discussions of issues before liaising with the professionals. These recommendations can be applied to all types of appointments where support staff encounters a professional. As people with ID, support staff and family caregivers often feel ignored by “professionals” or feel powerless to make changes or contribute to the decision about the prescription of psychotropic medication for people with ID or any other care planning (15, 16, 27, 28), EBCD will help them feel empowered and believe they have a “voice” to make meaningful contributions to the care of people with ID, including decisions about their medication.

In the following paragraphs, we have described how the co-design event’s main recommendations helped shape the SPECTROM module’s contents, “Effective liaison with professionals.”

### SPECTROM module on the effective liaison with professionals

The participants recommended gathering up-to-date information about the person with ID to inform professionals about the person with ID correctly. Support staff does not always know about a person’s history, possibly due to the shift work and a high staff turnover rate (29). For this reason, the SPECTROM module recommends the appointment of key support staff and describes their duties to ensure the same support staff is responsible for one individual with ID. This will help key support staff understand the person with ID and build a relationship of trust. Furthermore, we have also developed a resource called Yellow passport, which records all health-related information in an accessible format in one document that belongs to the person with ID and can be shared with professionals on a need-to-know basis. This will improve information sharing among professionals and agencies to improve the quality of care and make the right clinical decision at the right time.

The participants in the co-design event suggested creating a checklist for support staff to help gather patient-related information in preparation for the clinics/appointments. To comply with this recommendation, the SPECTROM training resource has developed an in-depth checklist for support staff to go through before appointments. Although the checklist is specifically focused on medication review, it can still be used for other appointments as it contains important information required for all appointments. The checklist includes gathering accurate information on (a) the current list of all medication and their doses, (b) any changes to medication doses since the last appointment, (c) medication side effects, (d) frequency of BtC and assessment results, (e) quality of life assessment, (f) opinions of the person with ID and their family caregivers, (g) records of neurodevelopmental, physical and/or mental health issues, (h) results of any examination and investigations and so on. The checklist will aid in gathering information about the person with ID and opinions of people with ID and their family caregivers regarding treatment options and/or medications.

Participants also suggested that people with ID often were not adequately informed about their meetings, treatment options, or medication information (17, 30, 31). Lack of information on appointments and procedures can lead to anxiety and impact the person with ID during appointments (32). Therefore, the SPECTROM module highlights the support staff’s responsibility to prepare the person with ID for multi-professional meetings using appropriate communication methods to involve the person fully. SPECTROM also provides videos on what happens during a health check, CT/MRI scan, blood tests, etc., to help prepare the person with ID for appointments. SPECTROM also provides hyperlinks to videos on various techniques that staff can train the person with ID on to help with their anxiety, such as relaxation exercises, mindfulness-based meditation etc.

Lack of communication and information sharing with the person with ID and their family caregivers has been highlighted in many studies (15–17, 27–33). Therefore, people with ID and other stakeholders resort to seeking information independently from a variety of sources such as medication leaflets, online/internet and training courses etc. (16). Professionals themselves have stated that they do not inform their patients who have ID as comprehensively as they would for patients without ID due to time constraints (34). Professionals are more likely to rely on family caregivers or support staff for information on people with ID (33, 34). Although professionals are aware of communication aids, both professionals and people with ID indicated that professionals often did not use them (29). They have also mentioned the lack of useful resources and tools, such as accessible information to pass on to people with ID and their caregivers, including staff, during appointments (33–35). Therefore, within SPECTROM resources, we have developed online freely downloadable accessible medication leaflets and provided links to useful, accessible resources to facilitate information exchange between the person with ID or other stakeholders and professionals. These accessible medication leaflets are updated versions of those developed 16 years ago (www.ld-medication.bham.ac.uk) and have since been emulated by many organisations in the UK and abroad. The SPECTROM module also provides techniques on how professionals can improve communication with persons with ID and support staff.

Professionals also spend a lot of time reviewing the documents rather than communicating with people with ID during appointments. Previous research has shown that doctors often prioritize making diagnostic and treatment decisions over communication during appointments due to a lack of time. Hence, to tackle this issue, in SPECTROM training, we recommended sharing critical documents/information before appointments so doctors and other professionals have more time to communicate with the person with ID during the appointments (29, 34). As per the suggestion from the co-design event, asking for information and critical documents from professionals before any appointment was highlighted as one of the responsibilities of support staff in SPECTROM. Participants in the co-design event suggested that professionals such as GPs and other doctors need the training to improve their communication with people with ID and/or ASD. This is also in line with previous research. According to professionals, there is a need for relevant training that can help improve the support they provide to people with ID (33–35). For this recommendation, we have hyperlinked a training resource for professionals on how they can improve the support they provide to individuals with ID.

As family caregivers and support staff play a pivotal role in the care provision of people with ID, information should be shared with them on a need-to-know basis. Professionals also acknowledge this, and research has shown they rely on family carers for communication to a great extent (33, 34). However, family caregivers often feel ignored and have not been included or involved in important decisions (15, 16, 27, 28). Family caregivers felt that they were not always asked for advice and that their views were not always sought (28, 29). Hence, the SPECTROM recommendation of holding an informal in-house appointment with all stakeholders, including the person with ID and their family caregivers, informs them about the contents that will be discussed in the formal appointment. SPECTROM module recommends regular meetings, including multi-professional team members with the support staff, the person with ID, and their family caregivers/advocates. Furthermore, as recommended in the SPECTROM module, the appointment of key support staff ensures that information from the formal appointment, such as action plans, is shared effectively with all those involved in the care of the person with ID.

### Adaptation of Experience Based Co-Design

We adapted the EBCD as the original phases of EBCD suggested by the toolkit could not fit within the project timescale. We also could not carry out some methods of capturing experiences as indicated by EBCD, such as observing or shadowing participants or filming interviews to understand the experiences of service users or staff, etc. It was too time-consuming and resource-intensive to observe participants or film interviews. SPECTROM used focus groups, meeting sessions, teleconferences, synthesis of existing evidence and a 1-day workshop to capture participants’ experiences and suggestions for the training programme. Eventually, a draft training programme was developed based on the recommendations. The SPECTROM intervention was successfully developed after many revisions and can be found on the SPECTROM website (https://spectrom.wixsite.com/project) (18).

Also, a field testing of SPECTROM on 20 trainees was successful and found significant results. Staff found SPECTROM helpful and empowering, evidenced by significantly improved scores on validated knowledge and attitude change scales and based on individual qualitative interview findings (18).

This paper shows that the EBCD approach can be successfully adapted and used to develop a staff training programme with stakeholder input in the development process. Other interventions have also been developed using this method but in different fields and targeting various health care services (6–8, 36). EBCD has also been adapted in multiple projects depending on project outcome and timescale, with the element of co-design event being used the most and the component of non-participant observation being used the least. Previous projects have used focus groups, workshops and surveys to capture stakeholder experience data similar to SPECTROM. Projects have also utilised modified EBCD to develop materials and resources rather than carry out service improvement (1). Developing an intervention while keeping stakeholders and service users at the heart of the development process will provide face validity and help make the intervention more relevant and practical for the audience and the purpose of the intervention. More researchers should utilise the EBCD approach to develop training programmes together collaboratively.

### Strengths

SPECTROM was developed using an MRC guideline and a modified EBCD method with stakeholder input from the beginning of the project. All stakeholders’ experiences and evidence of training programmes in neurodevelopmental disorders were used to develop SPECTROM. This allowed the training to be unique, acceptable, and relevant and helped to avoid duplication. All recommendations and action plans from the co-design event were clearly documented. It showed what information should be included in each module, what format this should take, and how to deliver it. Thus, SPECRTOM is replicable and auditable. Another strength is that there was no response and researcher bias as the participants worked on the activities independently, ensuring no influence from the researchers.

### Weaknesses

One weakness was that we could not use EBCD as suggested by the toolkit, and it had to be modified to fit our project timescale. For example, SPECTROM was unable to utilise videos to help capture experiences. However, we still had a successful and meaningful co-design session without it, where detailed information was gathered. Another weakness is that EBCD is resource-intensive, and projects need to ensure they have enough resources to carry out EBCD successfully. There was also some difficulty recruiting an adequate number of participants for the co-design event. However, in the end, we managed to recruit a sufficient number of participants representing various relevant stakeholder groups. On the day of the co-design event, five participants didn’t attend. Hence, not all tables/groups had a particular group of stakeholders to provide their input to the theme/topic. For the group involved in developing the content and format of effective liaison with professionals, only the trainer did not attend. Therefore, this group did not have the input of a trainer. The other drawback was the lack of direct participation of people with ID in the event, although their input was captured more effectively through their advisory group. Finally, it is possible that stakeholders involved in the study, such as support staff and service managers etc., are already motivated to reduce overmedication in people with ID, which may have introduced bias in the data.

## Conclusion

This paper has shown that a successful collaboration among key stakeholders can be achieved using a modified EBCD, and a training programme can be successfully developed. Although this is not the first paper that describes the use of EBCD to develop training materials, it is one of the first papers to develop a training programme for support staff to help reduce overmedication of people with ID using a modified EBCD and co-production. We have also shown that a modified EBCD can meaningfully gather experiences and suggestions to improve healthcare services as the original version of EBCD. In this paper, we describe the recommendations for the content, including case studies, for effective liaison with professionals’ module developed by all stakeholders collaboratively. Interdisciplinary collaboration is critical when attempting to improve health care services. More research aiming to improve healthcare services, whether through a training programme or intervention, should focus on service users’ experiences and utilise the EBCD approach.

## Data availability statement

The raw data supporting the conclusions of this article will be made available by the authors, without undue reservation.

## Author contributions

SD was the grant holder. BL analysed the co-design data. Both authors were involved in the conception and design of the study and in planning and holding the co-design event, contributed substantially to the preparation of the manuscript, and approved the final version of the manuscript.
