# Supplementary material for: Support staff liaising effectively with professionals for the rational use of psychotropics for behaviours that challenge in adults with intellectual disabilities: Findings from a co-design event
Source: Front Psychiatry. 2022 Sep 29;13:954522. doi: 10.3389/fpsyt.2022.954522 (PMC9559865; doi:10.3389/fpsyt.2022.954522)
Supplement: Supplementary file 1 [file Table_1.DOCX]

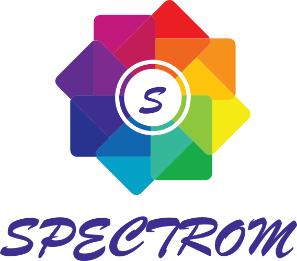
Group 5

We need your help to develop the modules for SPECTROM Psychoeducational Programme (PEP). You are given one hour to complete all these tasks. Do not worry if you cannot complete all tasks but please ensure you complete task 1 in one hour. If time allows you can try to complete other tasks in the order they are written.

Here is a guide (but not a prescription) for time management; (a) first 15 minutes: introduction and brain storming/ general discussion; (b) 30 minutes: content text/ case vignettes; (c) 10 minutes: rest of the tasks (if time allows); (d) 5 minutes: summarise main points/ scribe on flip chart for presentation.

Please use the paper to scribe the main points from your discussion. Please select a spokesperson from the group who will present the main findings to the whole group when you finished your task. Please write more detailed script if possible (over and above the main summary points for presentation), which you could either hand over to Bharati at the end of the session or email to us at a later date.

You will have five minutes to do the presentation, allowing for may be one or two questions/clarification from the floor.

There will be scope for further discussions at the end of the afternoon task session.

The PEP will have a written module and a face to face training session primarily geared towards service managers, senior care staff and those care staff who deal with severe challenging behaviour. The written modules could be accessed by family carers or CLDT members or any other stakeholders.

Therefore, please keep this audience in mind when preparing the contents of your theme. Therefore, we propose to produce basic modules and more detail/specific areas of modules to cater for different levels of knowledge and expertise.

Task 1

Your group is allocated the following theme.

**Effective liaison with GPs/ CLDT/ psychiatrist/ pharmacist**

We would like you to think about the contents that need to be included in this PEP module (what information will the care staff team need to effectively liaise with health professionals). Please feel free to add texts associated with each content if possible. Please also think of case vignettes to accompany appropriate content/text.

Some examples of the contents are;

“How can staff effectively communicate with GPs and other health professionals?”

“How can communication be maintained between care staff and GPs/ CLDT/ psychiatrist/ pharmacist?”

“What information should be given to GPs/ CLDT/ psychiatrist/ pharmacist during a visit to improve liaison?”

“How to develop a positive relationship with GPs/ CLDT/ psychiatrist/ pharmacist?”

*“How frequent should the reviews/meetings with health professionals be held?”*

*“What should be discussed by care staff team prior to meeting health professionals?”*

These are what some of our participants in the Focus Group said about liaising with GPs/ CLDT/ psychiatrist/ pharmacist:

*Service manager*

*“That why I say to them, if someone is attending a health appointment you’re coming too. If you’re their care worker, it’s not about me as the manager, sitting in that meeting and feeding back, you give your opinion. But I think before we go to that meeting, let’s have a discussion first so there’s no surprises. What are we going to say, what are we presenting to the clinical psychiatrist who doesn’t know this person as well as we do. What does the family members got to say about it? So we go into that meeting with that individual, if they’re open to input and they have the capacity to do that, to feed into that meeting so that it’s productive for that person.”*

*Trainer*

*“One of the things that we are seeing now, with our job, in transforming care, where somebody is moving, let’s say from the West Country into their local county, Essex where they grew up etc. So they’ve got a completely different team now in terms of care management, in terms of GP involvement, psychiatric involvement and that really has a big impact because if those people aren’t communicating with each other, from a very early onset stage, the staff have to put up with so many problems and issues.”*

*Service manager*

It’s about staff having that confidence to record that data. It’s not good enough to say someone had their breakfast, brushed their teeth. We want to know how they appeared that day, what was their behaviour like and given really good detail around that. Because that then feeds into any review meeting that they’re having, based on that medication. Um, as everyone has kind of hinted that the clinical psychiatrist may not know that person very well. You know we get a rotation of locum clinical psychiatrists and we’re very reliant on our support staff who are frontline staff, feeding into those review meetings.

Task 2

Once you have developed the main contents/texts/case vignettes, please think and describe how the contents of the module could be implemented in a day to day practice.

Task 3

Please rank the contents you have produced in terms of their hierarchy from basic to specific and detailed. So that we can decide what to include in a basic module and also in more specialised modules.

Task 4

Please think and describe how the written modules could be linked to the face to face training session (e.g., trainer to explain how to use the written module in the context of a real-life scenario, how to assimilate information from the written module, answer any question related to the module).

Task 5

If you have spare time, please feel free to contribute to the contents/case vignettes for other themes that were not originally allocated to you.
